# Simultaneous photoautotrophic production of DHA and EPA by *Tisochrysis lutea* and *Microchloropsis salina* in co-culture

**DOI:** 10.1186/s40643-022-00612-5

**Published:** 2022-12-19

**Authors:** Anna-Lena Thurn, Anna Stock, Sebastian Gerwald, Dirk Weuster-Botz

**Affiliations:** School of Engineering and Design, Chair of Biochemical Engineering, Technical University of Munich, Boltzmannstr. 15, 85748 Garching, Germany

**Keywords:** Docosahexaenoic acid (DHA), Eicosapentaenoic acid (EPA), *Microchloropsis salina*, *Tisochrysis lutea*, Co-cultivation, Photoautotrophic microalgae

## Abstract

Marine microalgae have received much attention as a sustainable source of the two health beneficial omega-3-fatty acids docosahexaenoic acid (DHA, C22:6) and eicosapentaenoic acid (EPA, C20:5). However, photoautotrophic monocultures of microalgae can only produce either DHA or EPA enriched biomass. An alternative may be the photoautotrophic co-cultivation of *Tisochrysis lutea* as DHA-producer with *Microchloropsis salina* for simultaneous EPA production to obtain EPA- and DHA-rich microalgae biomass in a nutritionally balanced ratio. Photoautotrophic co-cultivation processes of *T. lutea* and *M. salina* were studied, applying scalable and fully controlled lab-scale gas-lift flat-plate photobioreactors with LED illumination for dynamic climate simulation of a repeated sunny summer day in Australia [day–night cycles of incident light (PAR) and temperature]. Monocultures of both marine microalgae were used as reference batch processes. Differences in the autofluorescence of both microalgae enabled the individual measurement, of cell distributions in co-culture, by flow cytometry. The co-cultivation of *T. lutea* and *M. salina* in artificial sea water with an inoculation ratio of 1:3 resulted in a balanced biomass production of both microalgae simultaneously with a DHA:EPA ratio of almost 1:1 (26 mg_DHA_ g_CDW_^−1^, and 23 mg_EPA_ g_CDW_^−1^, respectively) at harvest after depletion of the initially added fertilizer. Surprisingly, more microalgae biomass was produced within 8 days in co-cultivation with an increase in the cell dry weight (CDW) concentration by 31%, compared to the monocultures with the same amount of light and fertilizer. What is more, DHA-content of the microalgae biomass was enhanced by 33% in the co-culture, whereas EPA-content remained unchanged compared to the monocultures.

**Graphical Abstract:**

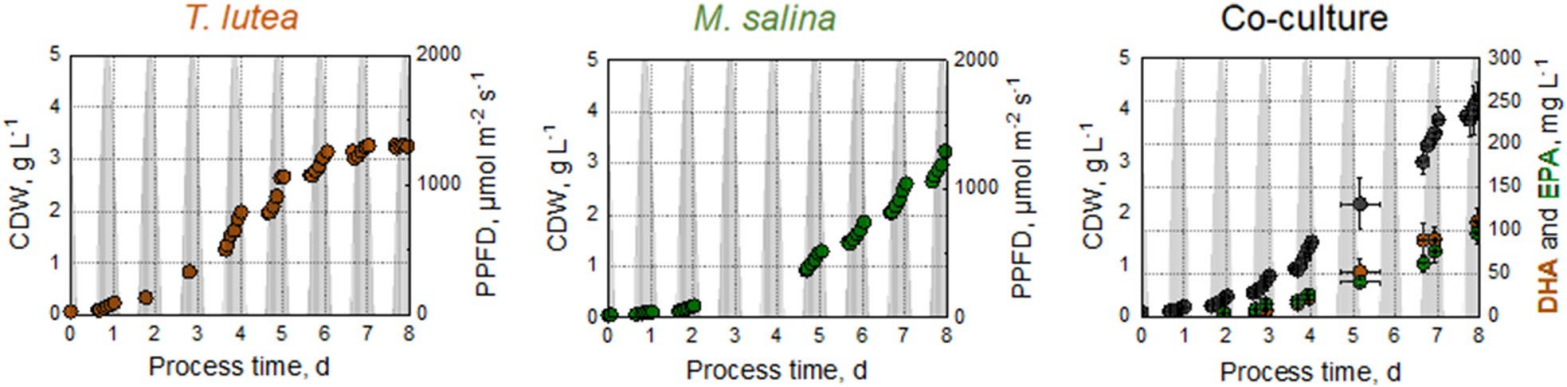

**Supplementary Information:**

The online version contains supplementary material available at 10.1186/s40643-022-00612-5.

## Introduction

Long chain polyunsaturated omega-3-fatty acids have been associated with many positive health effects due to their anti-inflammatory and cardio protective properties (Gogus and Smith 2010; Poudyal et al. 2011). In particular, docosahexaenoic acid (DHA, C22:6) and eicosapentaenoic acid (EPA, C20:5) play an essential role in the production of mediators, so called eicosanoids, that are important in regulating homeostatic functions (Cleland et al. 2003). By decreasing proinflammatory cytokines, lymphocytes proliferation and reactive oxygen species, the daily intake of DHA and EPA showed positive influence on the treatment and prevention of rheumatoid arthritis and coronary heart disease (Nielsen et al. 1992; Rennie et al. 2003; Volker et al. 2000). The recommended daily intake for a healthy human diet is a total of 250–500 mg DHA and EPA in a 1:1 ratio per day (Remize et al. 2021). A specific recommendation for an additional intake of 100–200 mg DHA per day applies for pregnant and lactating women (EFSA Panel on Dietetic Products and Allergies 2012).

DHA and EPA are synthesized in nature almost exclusively by marine microorganisms, especially microalgae, and are made available through fish and fish products, whereas terrestrial plants cannot produce these omega-3-fatty acids (Remize et al. 2021). Hence, microalgae are considered as primary producers of the marine food chain with regard to these fatty acids. Almost 90% of our omega-3-fatty acids supply comes from capture fisheries, often in form of fish oil supplements, as well as fishmeal and food fish (Tocher 2015). According to the FAO (2018), sustainable fish stocks have reached a plateau, having declined from 90% to 66.9% between 1974 and 2015, while the per capita consumption of fish and marine products has increased from 9 to 20 kg between 1961 and 2015. Consequently, aquaculture filled the increasing demand. Since fish cannot produce DHA and EPA by themselves, aquaculture also relies on fish oil and fish meal from capture fisheries (Tocher et al. 2019). Increasing water temperature due to global warming also is thought to affect the composition of lipids in phytoplankton and microalgae by reducing the unsaturated fraction of lipids (Remize et al. 2021). In conclusion, the decline in sustainable fish stocks, the increase in aquaculture and a rising demand for fish products limit the global supply of polyunsaturated fatty acids from traditional sources and demonstrate the importance of finding alternative omega-3-fatty acid sources (FAO 2018; Tocher et al. 2019).

One approach to solve the increasing gap between DHA and EPA availability through the marine food chain, and the need of a growing world population for the essential fatty acids, is the heterotrophic production of algal oils by microalgae or alga-like microorganisms (Hamilton et al. 2015; Sijtsma and de Swaaf 2004; Winwood 2013). Using carbon sources such as glucose or acetic acid, heterotrophic marine microorganisms such as *Schizochytrium* spp. and *Crypthecodinium cohnii* produce high amounts of PUFA’s and are currently used in commercial production (Barta et al. 2021; Sijtsma and de Swaaf 2004; Tocher et al. 2019). Another approach involves transgenic oilseed plants that express the corresponding genes from microalgae, so that the vegetable oil can be enriched with DHA and EPA (Napier et al. 2015).

The use of photoautotrophically produced marine microalgae biomass with adequate DHA and EPA contents, as a dietary supplement, represents an attractive non-GMO omega-3-fatty acid alternative (Adarme-Vega et al. 2012). Phototrophic microalgae can accumulate high amounts of lipids, including DHA and EPA and could, therefore, be a sustainable supply of these two health beneficial fatty acids (Ryckebosch et al. 2012). Further advantages for the photoautotrophic production of omega-3-fatty acids by microalgae are the use of saline water, no need of a carbon source or arable land and the capture of CO_2_ (Draaisma et al. 2013; Polishchuk et al. 2015; Schenk et al. 2008; Wang et al. 2008).

Promising marine microalgae for the intracellular accumulation of DHA are, e.g., *Isochyrsis galbana* or *Tisochrysis lutea,* accumulating 15–33 mg_DHA_ g_CDW_^−1^ (Aussant et al. 2018; Guihéneuf and Stengel 2013; Hu et al. 2018; Huerlimann et al. 2010). The marine golden-brown microalgal strains belong to the Haptophytes and are known for their use in aquaculture (Lin et al. 2007; Molina Grima et al. 1994). The genus *Nannochloropsis* (Eustigmatophyceae) is reported to accumulate high amounts of EPA with up to 50 mg_EPA_ g_CDW_^−1^ (Aussant et al. 2018; Chua and Schenk 2017; Gu et al. 2022; Hulatt et al. 2017; Polishchuk et al. 2015). In addition, the unicellular marine microalgae *Nannochloropsis* is already accepted as food (Chua and Schenk 2017) and can be cultivated outdoor on a large scale (Rodolfi et al. 2009; San Pedro et al. 2014). Unfortunately, most marine microalgae strains do not accumulate both fatty acids, but the supply of DHA and EPA for human nutrition is recommended to be in a balanced ratio of 1:1. Consequently, production of microalgal biomass via a monoculture would not provide a nutritionally balanced food supplement for human nutrition (Ryckebosch et al. 2014; Tocher et al. 2019).

Co-cultivation of two marine microalgae may provide an opportunity to expand the product range via a photoautotrophic process. Due to the imbalanced DHA and EPA ratio of marine microalgae, co-culturing an EPA-producer together with a DHA-producer may be an option to obtain microalgal biomass enriched with balanced amounts of DHA and EPA.

A synthetic co-culture of two phylogenetically different marine phototrophic microalgal strains has been recently demonstrated using *Tisochrysis lutea* (Haptophyte) and *Nannochloropsis oculata* (Eustigmatophyceae) (Maglie et al. 2021): Mono- and co-cultures were conducted in dimly illuminated (60 µmol m^−2^ s^−1^) shake flasks applying a day/night rhythm. Under identical process conditions, *N. oculata* showed analogous growth behaviour in co-culture compared to that in monoculture, whereas *T. lutea* grew significantly worse in co-culture, so that at the end of the photoautotrophic batch processes in illuminated shake flasks, *T. lutea* accounted for only 17% of the total biomass. Despite the low percentage of *T. lutea* in co-culture, the omega-3-fatty acid DHA was detectable, resulting in an omega-3-fatty acid combination of EPA and DHA in a ratio of 13:1 in the harvested biomass.

Other studies showed, in principle, the feasibility of synthetic microalgae co-cultures (Rashid et al. 2019; Tejido-Nuñez et al. 2020). Tejido-Nuñez et al. (2020) reported a co-culture of the two microalgae strains *Chlorella vulgaris* and *Tetradesmus obliquus* at a pilot scale in open thin-layer cascade photobioreactors under realistic outdoor conditions: using an inoculation ratio of 1:1, *C. vulgaris* accounted for only 10% of the final cells after a cultivation time of 29 days. A similar dominance effect of one species was reported by Zhao et al. (2014), when *Chlorella sp.* and *Monoraphidium sp*. were co-cultured in illuminated shake flasks. Nevertheless, the co-culture resulted in an enhanced lipid and biomass productivity. A co-culture of the two freshwater microalgae strains *Ettlia sp*. and *Chlorella sp.* achieved a higher biomass productivity compared to monocultures. Moreover, variation of the inoculation ratio resulted in an improved cell ratio of the two species in co-culture and further enhanced biomass productivity compared to 1:1 inoculated co-culture (Rashid et al. 2019). In contrast, Ishika et al. (2019) reported no increased biomass productivity and lipid content in a co-cultivation process of the two marine microalgae strains *Amphora sp.* and *Tetraselmis suecica*.

Except the synthetic co-culture reported by Tejido-Nuñez et al. (2020), all studies were conducted at lab scale and at constant and dim light irradiance. Due to high operational and energy costs of closed indoor photobioreactors, most commercial microalgae processes are realized in open pond systems, normally under realistic outdoor climate conditions (Borowitzka 1999). It has already been shown with photoautotrophic monocultures of microalgae that physical simulation of outdoor climate conditions (particularly dynamic incident light irradiation and temperature in day–night cycles) is essential in lab-scale studies to enable the scale-up to outdoor photobioreactors (Pfaffinger et al. 2019; Wolf et al. 2021).

As the co-cultivation of DHA-producing *Tisochrysis lutea* and EPA-producing *Nannochloropsis oculata* using dimly illuminated shake flasks scale did not result in a 1:1 DHA and EPA ratio (Maglie et al. 2021), and due to the fact that lab results may not easily scale to outdoor photobioreactors used on a commercial scale, we studied the photoautotrophic co-cultivation of the DHA-producing *Tisochrysis lutea* with the alternative EPA-producer *Microchloropsis salina* applying realistic physical climate simulation in the lab-scale photobioreactors. Strains from the genus *Microchloropsis* were formerly classified in the genus *Nannochloropsis* and, therefore, show strong genetic similarity (Fawley et al. 2015). Good EPA production (16 mg_EPA_ g_CDW_^−1^) have previously been reported for *Microchloropsis salina* under nitrogen replete conditions (Gu et al. 2022; Schädler et al. 2019).

In this work, we report on co-culture studies with the two marine phylogenetically different microalgal strains, *T. lutea* and *M. salina,* in artificial sea water for the photoautotrophic production of DHA and EPA balanced microalgae biomass in 1.8 L flat-plate gas-lift photobioreactors applying a dynamic climate simulation of a repeated sunny summer day in Australia. The temperatures ranged between 15 and 30 °C and the incident photon flux densities were varied between 0 and 2000 µmol m^−2^ s^−1^ to investigate biomass growth and omega-3-fatty acid production of both strains in monoculture and in co-culture with varying inoculation ratios in the batch processes.

## Materials and methods

### Microalgal strains, seed culture preparation and reaction media

The marine microalgae *Tisochrysis lutea* (CCMP 463) was obtained from the Provasoli-Guillard National Centre for Marine Algae and Microbiota (East Boothbay, USA). The marine microalgae *Microchloropsis salina* (SAG 40.85) was obtained from the Culture Collection of Algae at the University of Goettingen, Germany. A f/2 medium with modified levels of nitrogen (0.4 g L^−1^) and phosphor (45 mg L^−1^) in artificial seawater (ASW), at room temperature and laboratory light, in sterile shaking flasks was used for strain maintenance, for seed culture preparation as well as for all lab-scale batch processes. NaNO_3_ was used as the nitrogen source, while NaH_2_PO_4_ H_2_O served as the phosphate source. ASW was composed of NaCl (25 g L^−1^), MgCl_2_ (5.2 g L^−1^), NaSO_4_ (4.09 g L^−1^), CaCl_2_ (1.16 g L^−1^), KCl (0.695 g L^−1^), NaHCO_3_ (0.201 g L^−1^), KBr (0.101 g L^−1^), H_3_BO_3_ (0.027 g L^−1^) and NaF (0.003 g L^−1^) (“Sea Salt” ASTM D 1141–98, Lake Products Company, USA).

Preparation of seed cultures, as well as batch studies to identify appropriate inoculation ratios, were done in small bubble column reactors (250 mL) at a constant temperature of 25 °C, and a continuous irradiation of 83 ± 17 µmol m^−2^ s^−1^ in a Profors incubator (Infors, Bottmingen, Switzerland) with illumination (Havel et al. 2008). Mixing of the cultures was performed by applying a gas volume flow of 12 L h^−1^. CO_2_ was added in excess (2%, v/v).

### Flat-plate gas-lift photobioreactors operations and climate simulation

1.8 L flat-plate gas-lift photobioreactors (FPR, Labfors Lux 5, Infors HT, Bottmingen, Switzerland) equipped with a LED panel with 260 high performance LEDs, an illuminated surface area of 0.09 m^2^ and a light path way of 2 cm in the microalgae suspension were used for the photoautotrophic batch cultivation of microalgae in mono- and co-cultures. LEDs provided a characteristic spectrum in the visible range of light (400–800 nm). Optical sensors were used for pH and DO online measurement (Easyferm Plus ARC and VisiFerm DO ECS 120 H0, Hamilton Germany GmbH, Hoechst, Germany). A constant pH (pH 8.0) was maintained by controlled addition of CO_2_ (0–10%) at a constant gassing rate of 2 L min^−1^. Temperature was controlled using a temperature chamber located on the light-averted side of the FPR. For the inoculation of the flat-plate gas-lift reactors in the monoculture processes, seed cultures in the early stationary phase were used to achieve an initial OD_750_ of 0.2. For the co-culture processes, an inoculation ratio of 1:3 (*T. lutea*:*M. salina*) was used. Individual seed cultures of each microalgae strains served for the inoculation to obtain a total inoculation OD_750_ of 0.2. Monocultures were conducted as single experiments, co-culture cultivations were performed in triplicates.

**Fig. 1:**
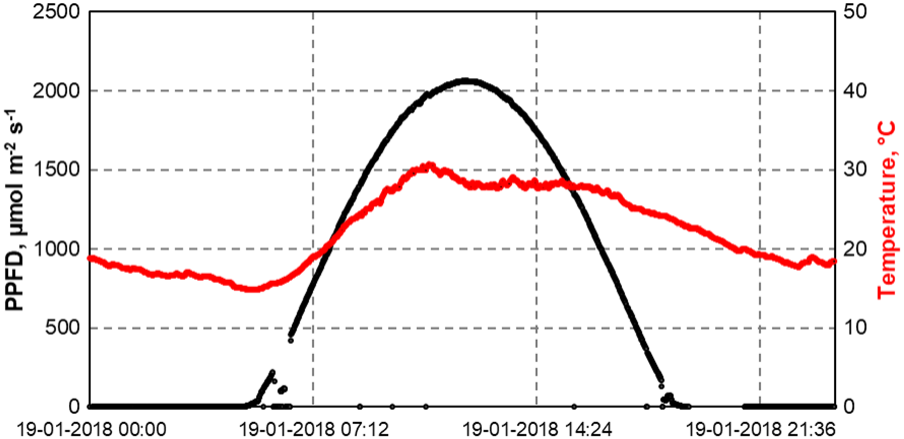
Solar photosynthetic photon flux density (PPFD) and air temperature. Data recorded in Newcastle, Australia, on January 19, 2018 by BSRN station no: 52. Data were provided by Duck (2018) (BSRN). All batch processes in flat-plate gas-lift photobioreactors were performed under these repeatedly applied conditions

Large scale outdoor microalgae production depends on locations that ensure a suitable climate for microalgae growth. To simulate realistic outdoor climate conditions, a physically dynamic climate simulation with varying temperatures and varying photon flux densities was used for all batch processes in flat-plate gas-lift reactors. The selected day for physical dynamic climate simulation was January 19, 2018 in Newcastle, Australia (Fig. 1) (Duck 2018). The Australian summer day with high solar irradiation of up to 2000 µmol m^−2^ s^−1^ and temperatures up to 30 °C during the day (15 °C at night) was repeated daily for all batch processes.

### Determination of cell ratio in co-culture

**Fig. 2:**
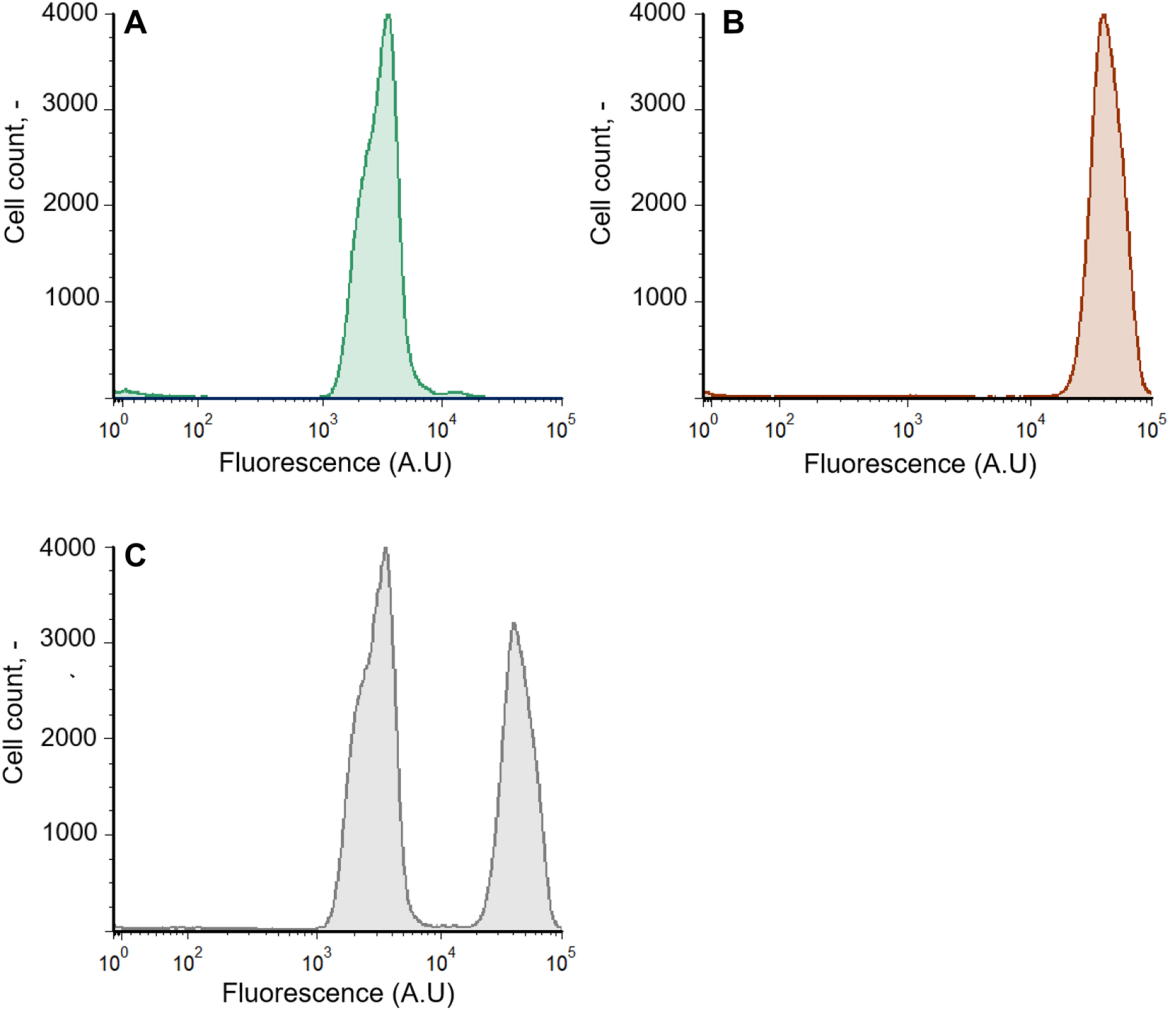
Histograms of three samples with **A** 100% *M. salina*, **B** 100% *T. lutea* and **C** mixed sample containing both algal strains at an OD_750_ of 0.2. *X*-axis shows the intensity of the fluorescence signal excited by a red laser at 640 nm and detected using the 660/10 nm bandpass filter. *Y*-axis shows the cell counts of the algae strains

Autofluorescence of microalgae enabled the differentiation of *M. salina* and *T. lutea* in co-culture. Determination of individual cell counts was conducted using a flow cytometer (BD FACS Melody Cell Sorter; BD Biosciences, San Jose, CA, USA). Analysis was performed with BD FACSChorus software (BD Bioscience). The event rate was set to 1000 events per second. Cell samples were excited by a red laser at 640 nm and the emission was measured by applying the 660/10 nm bandpass filter. Hereby, the fluorescence intensity of the microalgae strain *T. lutea* was higher, which allowed the separation of the two algae strains (Fig. 2). Fluorescence signals were plotted as histograms with cell counts vs. fluorescence intensity in bi-exponential display. Specific linear correlation factors between cell counts and OD_750_ were determined prior to co-culture experiments by examining the cell counts in manually mixed samples with different ratio combinations of both strains characterized by their OD_750_. All samples were diluted with 25 g L^−1^ NaCl to obtain an OD_750_ of 0.2.

### Determination of optical density and cell dry weight

The optical density (OD_750_) of the microalgae in suspension was measured in triplicates using a UV–Vis-Spectrometer (Genesys 10 UV–Vis, Thermo Fischer, Waltham) using tap water with 25 g L^−1^ NaCl as a blank. The cell dry weight was measured gravimetrically in triplicates by loading a defined volume of the microalgae culture on a pre-dried and weighted glass microfiber filter (GF/C, Whatman, GE Healthcare). Loaded filters were washed with deionized water, dried 48 h at 80 °C to obtain mass constancy and were weighed again. The differences in weight between loaded and unloaded filter were used to estimate the cell dry weight.

### Determination of nitrate concentration and salinity

A colorimetric enzyme assay (Nitrite/Nitrate colorimetric method, Roche Diagnostics GmbH, Penzberg, Germany) was used for the determination of the nitrate concentration. A volume of 1 mL of the sample was first centrifuged at 13,000 rpm and the resulting supernatant was diluted with double-distilled water to a concentration of 0.05–0.5 g L^−1^. Nitrate concentration was measured photometrically based on the reduction of nitrate to nitrite by the enzyme nitrate reductase.

Salinity of the cultivation media was measured using a refractometer (Hanna Instruments, Deutschland GmbH, Vöhringen, Germany) determining the refractive index of the liquid.

### Determination of absorption spectra of fucoxanthin and chlorophyll

For the pigment extraction, samples of 5 mL were centrifuged at 3000 rpm for 20 min. The supernatant was discarded and the resulting pellet was resuspended in acidified 90% acetone (containing 0.075 M HCl) and incubated for 24 h at 4 °C in the dark. Acidification of the solvent allows the differentiation between chlorophyll a and pheophytin (Lorenzen, 1967). The centrifugation step was repeated and the supernatant was used for recording the absorbance spectra between 350 and 750 nm in 1 nm steps. To analyse the absorbance spectra, standards of fucoxanthin and chlorophyll a were dissolved in acidified acetone and absorption spectra of these reference substances were also recorded.

### Determination of total lipid content and DHA and EPA concentration

To determine the total lipid, DHA, and EPA content, 10–25 mL of each sample were frozen at − 80 °C and then lyophilized at − 50 °C and 0.12 mbar for 48 h (Lyophilizer Alpha 1–2 LD plus, Martin Christ Gefriertrocknungsanlagen GmbH, Osterode am Harz, Germany). Subsequently, depending on the biomass concentration and salinity, 5 mg of pure biomass was weighed and transferred to analysis vials. To obtain a defined amount (mg) of pure biomass, the amount of weighed lyophylisied algae powder varied at each sampling time depending on to the actual ratio of salt and biomass concentration in the media. For transesterification and extraction, a method by Griffith et al. (2010), was conducted as described previously (Pfaffinger et al. 2016). Separation of FAMES was performed with a gas chromatrograph (GC-2010, Shimadzu, Kyoto; Japan) with a fused silica FAMEWAX column (Restek GmbH, Bad Homburg, Germany) and a flame ionization detector, according to their chain length, using a temperature gradient (120 °C up to 220 °C with 7 °C min^−1^, 220 °C for 20 min). Helium with a flow rate of 3 mL min^−1^ served as carrier gas. The injector temperature was 220 °C, while the flame ionization detector temperature was 250 °C. A marine oil mix (Marine Oil FAME Mix, C14:0 to C24:1) was used as an external standard to identify the peaks. Due to the fact that microalgae do not produce C_12_ fatty acids (Metting 1996), C12-TAG (Sigma-Aldrich, Saint Louis, USA) was selected as an internal standard. Total lipid content was calculated using the sum of all peaks (excluding the internal standard) normalised to the amount of biomass extracted. DHA and EPA concentration were determined by calculating the proportion of their area to total peak area including their individual response factor. EPA and DHA content (mg g_CDW_^−1^) was calculated relating the FA concentration to the actual cell dry weight.

## Results and discussion

### Photoautotrophic monoculture

**Fig. 3:**
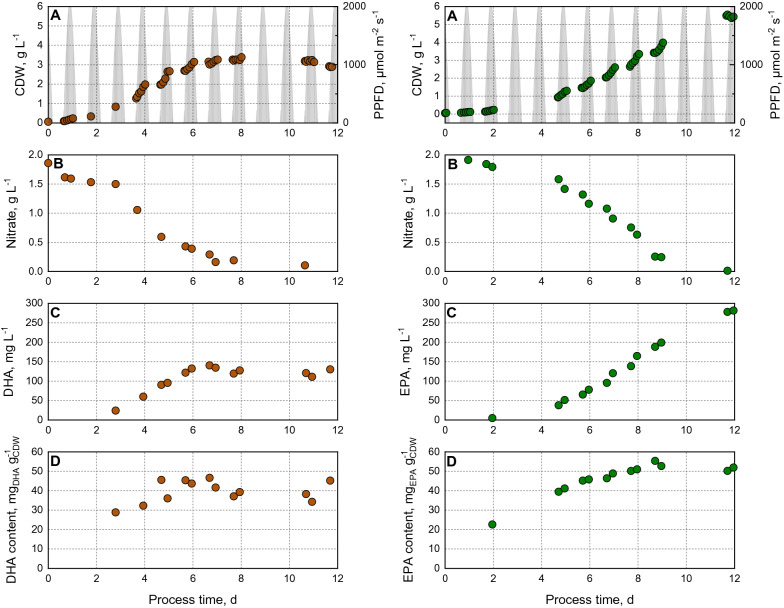
Phototrophic batch processes with *T. lutea* (right, red circle) and *M. salina* (left, green circle) in flat-plate gas-lift photobioreactors under simulated climate conditions. **A** Cell dry weight concentration, **B** nitrate concentration, **C** DHA (red circle) or EPA (green circle) concentration, and **D** DHA (red circle) or EPA (green circle) content of total cell dry weight. The batch processes were operated at a working volume of 1.8 L, pH 8.0 and an initial nitrate supply of 1.8 g L^−1^. Monocultures were inoculated at an OD_750_ of 0.2

Batch processes with either the microalgae *T. lutea* or *M. salina* were performed in flat-plate gas-lift photobioreactors applying incident light and temperature profiles of a repeated Australian summer day (Fig. 3). Biomass formation, nitrate consumption, as well as DHA and EPA production were monitored. The cultivations were stopped after 12 days. Regarding the process with *T. lutea*, the microalgae strain reached the stationary growth phase with a final CDW concentration of ~ 3 g L^−1^ after a process time of 7 days despite a residual nitrate concentration of about 0.2 g L^−1^ in the media. Hence, 90% of the initial supplied nitrate was consumed, suggesting that a higher nutrient concentration would not lead to higher biomass production. Due to the dark colour of this microalgae and the resulting self-shading effects at high cell densities, light limitation may have been the reason for no further growth.

The DHA concentration increased, with increasing biomass and decreasing nitrate concentration, and reached a final concentration of 124 ± 8 mg L^−1^ after 7 days, when biomass growth and nitrate consumption stopped. DHA accumulation of *T. lutea* was observed to be growth coupled under nitrogen replete conditions. A stationary DHA content of 40 ± 4 mg_DHA_ g_CDW_^−1^ was already obtained after 5 days of cultivation. Sun et al. (2019) had shown increased DHA productivity and DHA content under nitrogen replete conditions demonstrating a growth-related DHA accumulation with *T. lutea*. Hu et al. (2019) reported a maximal DHA content of 27.5 mg g^−1^ under nitrogen replete conditions and a decreased DHA production under nitrogen starved conditions. The proportion of the fatty acid DHA remained constant over the entire process at around 10% of the total fatty acids (TFA), what is consistent with data reported in literature (Aussant et al. 2018; Lin et al. 2007; Rasdi and Qin 2015).

The batch process with *M. salina* resulted in a maximal final biomass concentration of 5.5 g L^−1^ after the total initial nitrate has been consumed. From day 4 until the end of the batch process on day 12, *M. salina* remained in the linear growth phase. Regarding the production of omega-3-fatty acids, only EPA was detected, DHA was not produced, as expected. Proportionally to the decreasing nitrate concentration and increasing biomass concentration, the EPA content increased to a maximum of 30% of the TFA. When nitrogen-limited conditions prevailed, the EPA fraction in the TFA began to decrease (data not shown). Comparable to the DHA production with *T. lutea*, EPA was formed in a growth-related manner under nutrient-saturated conditions. A maximal EPA concentration of 280 mg L^−1^ at the end of the process and a stationary EPA content of 52 ± 2 mg_EPA_ g_CDW_^−1^ was reached after 8 days. At sodium nitrate fed-batch operation mode, Gharat et al. (2018) reported a higher EPA concentration of 168 mg L^−1^ and an EPA content of 33% of the TFA. Gu et al. (2022) achieved an EPA content of 16 mg_EPA_ g_CDW_^−1^ in 500 mL Erlenmeyer flasks with *M. salina*. A comparable EPA content of 49.3 mg g^−1^ was reached with *Nannochloropsis* sp. in 400 mL flat panel reactors after a cultivation time of 12 days (Hulatt et al. 2017). Similar microalgae growth was observed before using incident light photon flux density dynamics (PFD) very similar to this study in flat-plate gas-lift photobioreactors (Pfaffinger et al. 2016, 2019).

### Study of the inoculation ratio in co-culture

In the early exponential growth phase in monoculture, *T. lutea* showed a daily growth rate almost twice as high as of *M. salina*. Moreover, *T. lutea* already reached a steady-state biomass concentration after about 7 days, while *M. salina* was still in linear growth until the end of the process after 12 days and thus achieved a higher final CDW concentration. Both algal strains accumulated DHA and EPA under nutrient-saturated conditions. Several studies showed an increase of PUFA in marine microalgae when cultivated in nitrogen-replete media (Adarme-Vega et al. 2012; Gharat et al. 2018; Kim et al. 2016; Schädler et al. 2019). PUFAs facilitate maintenance of fluidity of the membrane what is required during cell division and growth under optimized nitrogen concentrations for growth (Adarme-Vega et al. 2012; Gharat et al. 2018).

Due to different growth kinetics, co-cultivation with an inoculation ratio of 1:1 would probably result in a dominance of *T. lutea* in the photobioreactor. Based on the data of the monocultures, *T. lutea* and *M. salina* should be present in an approximately 1:1 ratio at harvest time in the photobioreactor, because the omega-3-contents in the biomass of the monocultures were comparable (40 ± 4 mg_DHA_ g_CDW_^−1^, and 52 ± 2 mg_EPA_ g_CDW_^−1^).

**Fig. 4:**
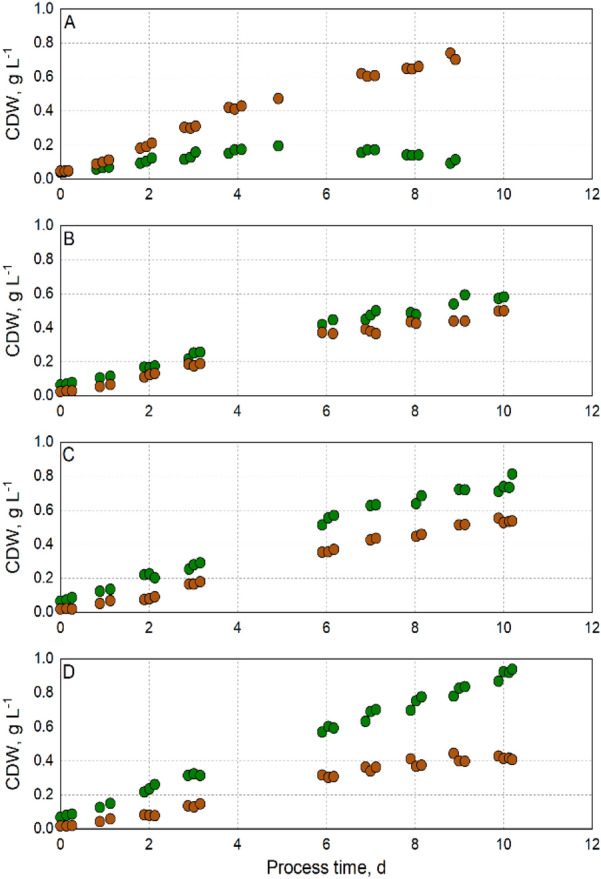
Variation of inoculation ratios in phototrophic batch processes with *T. lutea* (red circle) and *M. salina* (green circle) in co-culture at constant light irradiance of 83 ± 17 µmol m^−2^ s^−1^ and a constant temperature of 25 °C in 200 mL bubble column reactors operated in parallel. **A** Inoculation ratio 1:1, **B** inoculation ratio 1:2, **C** inoculation ratio 1:3, **D** inoculation ratio 1:4 (*T. lutea*:*M. salina*). The batch processes were operated at a working volume of 200 mL and pH 8.0

To search for a balanced ratio of *T. lutea* and *M. salina* in co-culture at harvest time, photoautotrophic batch processes with varying inoculation ratios of *T. lutea* and *M. salina* (1:1; 1:2; 1:3, and 1:4, respectively) were studied in parallel bubble column reactors at constant light irradiation and temperature (Fig. 4). The pH was controlled manually. As expected, an inoculation ratio of 1:1 led to the dominance of *T. lutea* after 2 days of the batch process, while an inoculation ratio of 1:4 resulted in approximately twofold higher cell dry weight concentrations of *M. salina* during the batch process. Similar cell dry weight concentrations of both algal strains in co-culture were obtained with inoculation ratios of 1:2 or 1:3 due to the different growth kinetics of the two microalgal strains.

### Phototrophic co-culture of *T. lutea *and *M. salina* in flat-plate gas-lift reactors

**Fig. 5:**
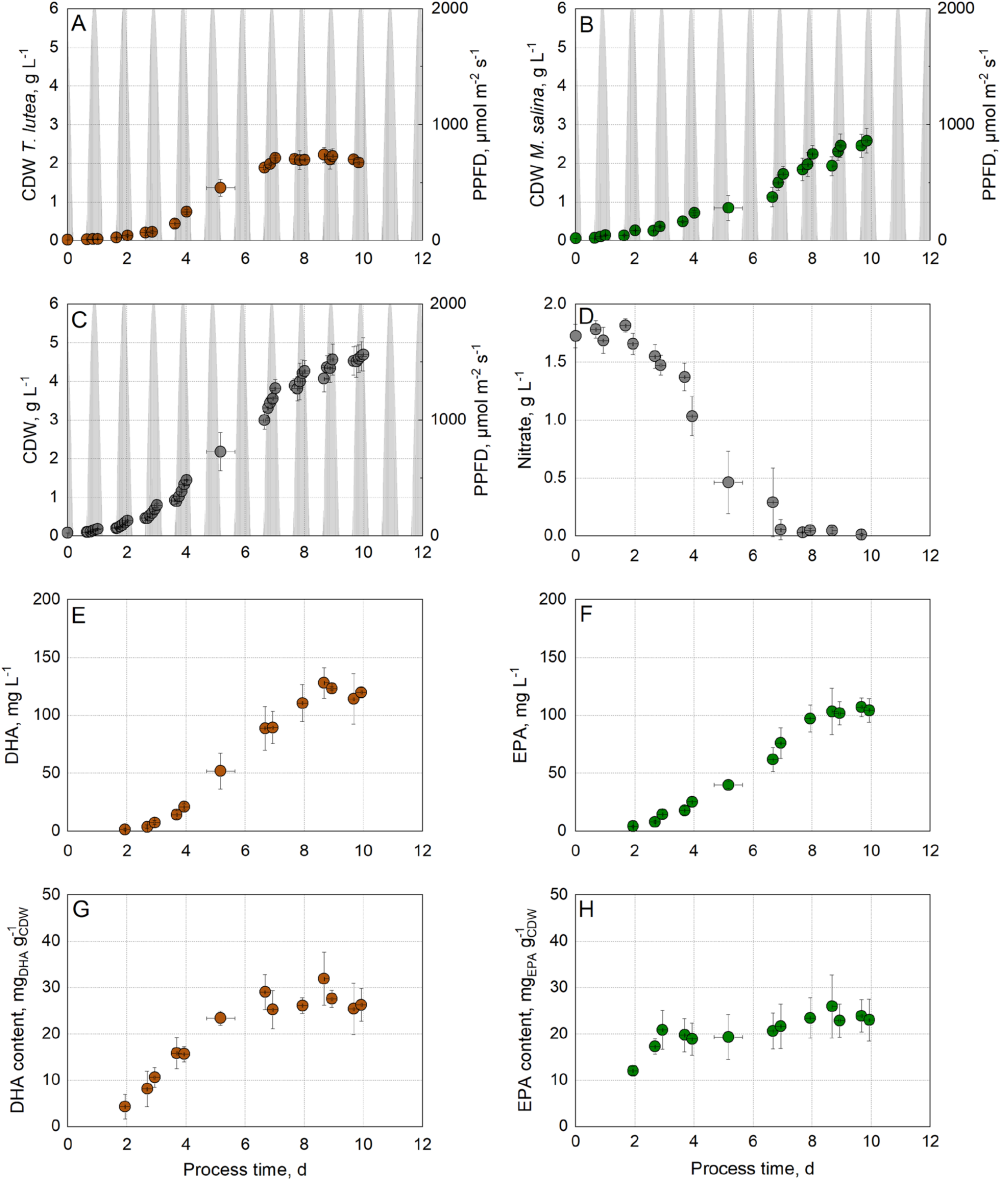
Phototrophic batch process with *T. lutea* (red circle) and *M. salina* (green circle) in co-culture with an inoculation ratio of 1:3 in flat-plate gas-lift photobioreactors under simulated climate conditions. **A** cell dry weight concentration of *T. lutea*, **B** cell dry weight concentration of *M. salina*, **C** total cell dry weight concentration, **D** nitrate concentration, **E** DHA concentration, **F** EPA concentration, **G** DHA content of total cell dry weight, **H** EPA content of total cell dry weight. The batch process was operated at a working volume of 1.8 L, pH 8.0 and an initial nitrate supply of 1.8 g L^−1^. Data are expressed as a mean ± SD (*n* = 3)

Autophototrophic co-culture of *T. lutea* and *M. salina* was performed in fully controlled flat-plate gas-lift photobioreactors applying a physical climate simulation of a repeated Australian sunny summer day. The same feed medium concentration was used as in monocultures. An inoculation ratio of 1:3 (*T. lutea*:*M. salina*) was chosen first (Fig. 5).

*T. lutea* reached the stationary growth phase with a cell dry weight concentration of 2.1 ± 0.1 g L^−1^ on days 7–8 when the initially supplied nitrate has been consumed. *M. salina* achieved a CDW concentration of 2.2 ± 0.2 g L^−1^ after 8 days and a final CDW concentration of 2.6 ± 0.3 g L^−1^ on day 10. The total CDW concentration increased until day 10 of the batch process up to 4.7 ± 0.4 g L^−1^. A balanced ratio of *T. lutea* and *M. salina* regarding the cell dry weight concentrations was achieved over the whole process time. The growth behaviour of both algal strains was comparable to that in monoculture. Furthermore, both omega-3-fatty acids DHA and EPA were accumulated under nitrogen saturated conditions as shown before in monocultures. Therefore, an optimal harvest time of the co-culture would be at day 8, when the initially added nitrate has been consumed totally.

Nitrogen deficiency at a process time of 7–8 days resulted in stationary DHA and EPA concentrations of 119 ± 6 mg L^−1^, and 102 ± 3 mg L^−1^, respectively. The DHA content in co-culture reached a steady state of 27 ± 2 mg_DHA_ g_CDW_^−1^ at the beginning of the stationary growth phase of *T. lutea*. In monoculture, DHA content already remained constant in the linear growth phase (from day 6), while nitrogen was still present in the reactor (Fig. 3). The EPA content in the microalgal biomass increased only during the first 3 days of the batch process and then remained constant at 22 ± 2 mg_EPA_ g_CDW_^−1^. In the monoculture of *M. salina*, the EPA content increased until day 9 (Fig. 3).

Co-cultivation of *T. lutea* and *M. salina* with an inoculation ratio of 1:3 with twice the initial supply of fertilizer (nitrate, phosphate, trace elements and vitamins) did not result in an enhanced final CDW concentration or DHA and EPA production (Additional file 1).

Compared to the co-culture described in Maglie et al. (2021) with *T. lutea* and *N. oculata*, in this co-cultivation study, the EPA content was 30% higher and the DHA content of the microalgae biomass was ≈ 20 times higher (harvesting on day 8, after nitrogen depletion). Moreover, an inoculation ratio of 1:3 (*T. lutea*:*M. salina*) allowed similar biomass concentrations of the two microalgal strains to be obtained over the entire process time of the batch process. After 8 days, each of the microalgal strains, *M. salina* and *T. lutea* had achieved above 2 g_CDW_ L^−1^. Moreover, both omega-3 fatty acids were formed in a balanced ratio of almost 1:1 in this co-cultivation process, thus demonstrating a simple and efficient new process for the production of DHA and EPA containing microalgae biomass in a nutritionally optimal ratio.

The comparison of two photoautotrophic monocultures (*T. lutea* and *M. salina*) with the co-cultivation of both strains in defined flat-plate gas-lift photobioreactors with identical physical climate simulation showed clear advantages of the co-cultivation process with respect to the same illuminated photobioreactor surface (e.g., operating two identical photobioreactors each with a monoculture of *T. lutea* or *M. salina* and mixing the both algal suspensions after harvest, compared to two identical photobioreactors with a co-culture of *T. lutea* and *M. salina*): after a process time of 8 days (harvest time after nitrate depletion), biomass production in co-culture was increased by 31 ± 7% (4.2 ± 0.3 g L^−1^ instead of 3.2 g L^−1^ in the monocultures). In the monoculture of *M. salina*, only 37% of the initially supplied nitrate was consumed, while in co-culture almost the entire nitrate concentration was used. Thus, the nutrient usage was clearly enhanced in co-culture (97 ± 2.6%). Co-cultivation led to an increase in the DHA concentration by 73 ± 14% (111 ± 16 mg L^−1^ instead of 64 mg L^−1^). DHA content of the microalgae biomass in co-culture was enhanced by 33 ± 8% (26 ± 2 mg_DHA_ g_CDW_ instead of 19.5 mg_DHA_ g_CDW_). In contrast, EPA concentration and EPA content remained unchanged within the estimation error compared to the mixing of both monocultures (Table 1).

**Table 1:** Comparison of photoautotrophic monocultures of the DHA-producing microalgae *T. lutea* and the EPA-producing *M. salina* with a co-cultivation of the two microalgae at a harvest time after 8 days (consumption of initially supplied nitrate in co-culture) relative to the illuminated surface of the flat-plate gas-lift photobioreactors

	*T. lutea*	*M. salina*	Co-culture	Co-culture
CDW (g L^−1^)	3.2	3.2	4.2 ± 0.3	+ 31 ± 7%
Nitrate usage (%)	90	37	97 ± 2.6	–
DHA (mg L^−1^)	64	0	111 ± 16	+ 73 ± 14%
DHA (mg_DHA_ g_CDW_)	19.5	0	26 ± 2	+ 33 ± 8%
EPA (mg L^−1^)	0	82.5	97 ± 12	+ 18 ± 12%
EPA (mg_EPA_ g_CDW_)	0	25.5	23 ± 4	–

**Fig. 6:**
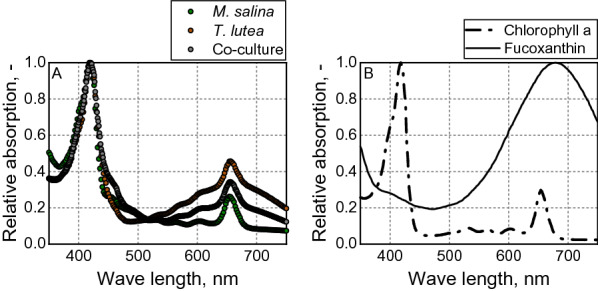
**A** Absorption spectra of monocultures of *M. salina* (green dashed lines) and *T. lutea* (red dashed lines) and of a co-culture (green dashed lines) of both microalgae after acid acetone extraction. **B** Absorption spectra of the pigments Chlorophyll a (dashed dots) and Fucoxanthin (dashed line) after acid acetone extraction

The increased biomass concentration in co-culture could be due to an enhanced utilization of the light spectrum. The two utilized marine microalgae strains are phylogenetically clearly different (Slocombe et al. 2015), which can have an impact on light spectrum utilization. The absorbance spectra of the two algal strains as well as the co-culture revealed clear differences. *T. lutea* showed higher absorbance than *M. salina* in the region above 550 nm. This effect could also be seen in the co-culture (Fig. 6). The additional absorption of the *Tisochrysis lutea* extract is most likely due to the carotenoid fucoxanthin, as the reference measurements of the absorbance spectra with pure substances after acid acetone extraction demonstrated. The extraction of the carotenoid fucoxanthin, which is produced solely by *T. lutea*, leads to a shift of the absorption spectrum in the extract, with increased absorption between 550 and 750 nm and a maximum at 678 nm instead of a maximum at 450 nm (Riemann 1978). In the literature, fucoxanthin, in addition to the generally known photoprotective properties, is also attributed an important role in the light reaction of photosynthesis. Together with chlorophyll and other proteins, the carotenoid forms a complex located in the thylakoids, whereby light of the green range can also be absorbed (Chen et al. 2022; Kuczynska et al. 2015). *Tisochrysis lutea* might thus be able to photosynthetically use light at wavelengths that cannot be used by *Microchloropsis salina*, which could be a possible reason for the higher biomass yield in co-culture compared to the monoculture.

An increased biomass yield of microalgae in co-culture has already been reported recently. Rashid et al. (2019) observed an increased biomass concentration in a photoautotrophic co-culture of the freshwater microalgae strains *Chlorella sp.* and *Ettlia sp.* compared to their monocultures in illuminated bubble column reactors. They assumed a symbiotic, growth-stimulating relationship of the two microalgae strains in co-culture.

## Conclusion

Photoautotrophic co-cultivation of the two phylogenetically different marine microalgae *Tisochrysis lutea* and *Microchloropsis salina* in flat-plate gas-lift photobioreactors enables the production of microalgae biomass with well-balanced DHA and EPA content after 8 days in batch processes, applying a physically dynamic climate simulation of a repeated sunny summer day and night in Australia. Choosing the right inoculation ratio is important to achieve a well-balanced biomass yield of both species, as well as a well-balanced DHA and EPA content in the microalgae biomass at harvest time. Physical simulations of realistic outdoor conditions in lab-scale studies are necessary to ensure an easy scale-up of microalgae production processes to outdoor photobioreactor systems as already shown before (Pfaffinger et al. 2019; Wolf et al. 2021). Increased biomass concentration in the co-culture compared to the corresponding monocultures of both microalgae may be caused by an improved utilization of light at different wavelengths by the two phylogenetically different marine microalgae, but needs further research. The photoautotrophically produced marine microalgae biomass enriched with DHA and EPA in a well-balanced ratio represents an attractive non-GMO omega-3-fatty acid alternative to marine fish, if the (salty) microalgae biomass can be used as a dietary supplement for human nutrition after drying.

### Supplementary Information

**Additional file 1: Fig. S1. **Phototrophic batch process with T. lutea (●) and M. salina (●) in co-culture with an inoculation ratio of 1:3 in flat-plate gas-lift photobioreactors under simulated climate conditions. (A) cell dry weight concentration of T. lutea, (B) cell dry weight concentration of M. salina, (C) total cell dry weight concentration, (D) nitrate concentration, (E) DHA concentration, (F) EPA concentration, (G) DHA content of cell dry weight, (H) EPA content of cell dry weight. The batch process was operated at a working volume of 1.8 L, pH 8.0 and an initial nitrate supply of 3.6 g L-1.

## Data Availability

All data generated or analysed during this study are included in this published article.

## Abbreviations

CDW: Cell dry weight
DHA: Docosahexaenoic acid
EPA: Eicosapentaenoic acid
FAME: Fatty acid methyl ester
FPR: Flat-plat gas-lift photobioreactor
PPFD: Photosynthetic photon flux density
PUFA: Polyunsaturated fatty acids
TAG: Triacylglyceride

## References

[CR1] Duck B (2018) Basic measurements of radiation at station Newcastle (2017–11) Retrieved from: 10.1594/PANGAEA.896530

[CR2] Adarme-Vega TC, Lim DKY, Timmins M, Vernen F, Li Y, Schenk PM (2012). Microalgal biofactories: a promising approach towards sustainable omega-3 fatty acid production. Microb Cell Fact.

[CR3] Aussant J, Guihéneuf F, Stengel DB (2018). Impact of temperature on fatty acid composition and nutritional value in eight species of microalgae. Appl Microbiol Biotechnol.

[CR4] Barta DG, Coman V, Vodnar DC (2021). Microalgae as sources of omega-3 polyunsaturated fatty acids: biotechnological aspects. Algal Res.

[CR5] Borowitzka MA (1999). Commercial production of microalgae: ponds, tanks, tubes and fermenters. J Biotechnol.

[CR6] Chen D, Yuan X, Zheng X, Fang J, Lin G, Li R, Xue T (2022). Multi-omics analyses provide insight into the biosynthesis pathways of fucoxanthin in *Isochrysis galbana*. Genom Proteom Bioinform.

[CR7] Chua E, Schenk P (2017). A biorefinery for Nannochloropsis: induction, harvesting, and extraction of EPA-rich oil and high-value protein. Biores Technol.

[CR8] Cleland LG, James MJ, Proudman SM (2003). The role of fish oils in the treatment of rheumatoid arthritis. Drugs.

[CR9] Draaisma RB, Wijffels RH, Slegers PM, Brentner LB, Roy A, Barbosa MJ (2013). Food commodities from microalgae. Curr Opin Biotechnol.

[CR10] EFSA Panel on Dietetic Products, N., & Allergies (2012). Scientific opinion on the tolerable upper intake level of eicosapentaenoic acid (EPA), docosahexaenoic acid (DHA) and docosapentaenoic acid (DPA). EFSA J.

[CR11] FAO FAAO (2018) The state of world fisheries and aquaculture 2018

[CR12] Fawley MW, Jameson I, Fawley KP (2015). The phylogeny of the genus *Nannochloropsis* (Monodopsidaceae, Eustigmatophyceae), with descriptions of *N. australis* sp. Nov. and *Microchloropsis *gen. nov.. Phycologia.

[CR13] Gharat K, Agarwal A, Pandit RA, Lali AM (2018). Development of fed batch strategies to improve the production of eicosapentaenoic acid from a marine microalga *Nannochloropsis oculata*. Bioresour Technol Rep.

[CR14] Gogus U, Smith C (2010). *n* − 3 Omega fatty acids: a review of current knowledge. Int J Food Sci Technol.

[CR15] Gu W, Kavanagh JM, McClure DD (2022). Towards a sustainable supply of omega-3 fatty acids: screening microalgae for scalable production of eicosapentaenoic acid (EPA). Algal Res.

[CR16] Guihéneuf F, Stengel DB (2013). LC-PUFA-enriched oil production by microalgae: accumulation of lipid and triacylglycerols containing *n* − 3 LC-PUFA is triggered by nitrogen limitation and inorganic carbon availability in the marine haptophyte *Pavlova lutheri*. Mar Drugs.

[CR17] Hamilton ML, Warwick J, Terry A, Allen MJ, Napier JA, Sayanova O (2015). Towards the industrial production of omega-3 long chain polyunsaturated fatty acids from a genetically modified diatom *Phaeodactylum tricornutum*. PLoS ONE.

[CR18] Havel J, Franco-Lara E, Weuster-Botz D (2008). A parallel bubble column system for the cultivation of phototrophic microorganisms. Biotechnol Lett.

[CR19] Hu H, Ma L-L, Shen X-F, Li J-Y, Wang H-F, Zeng RJ (2018). Effect of cultivation mode on the production of docosahexaenoic acid by *Tisochrysis lutea*. AMB Express.

[CR20] Hu H, Li JY, Pan XR, Zhang F, Ma LL, Wang HJ, Zeng RJ (2019). Different DHA or EPA production responses to nutrient stress in the marine microalga *Tisochrysis lutea* and the freshwater microalga *Monodus subterraneus*. Sci Total Environ.

[CR21] Huerlimann R, de Nys R, Heimann K (2010). Growth, lipid content, productivity, and fatty acid composition of tropical microalgae for scale-up production. Biotechnol Bioeng.

[CR22] Hulatt CJ, Wijffels RH, Bolla S, Kiron V (2017). Production of fatty acids and protein by nannochloropsis in flat-plate photobioreactors. PLoS ONE.

[CR23] Ishika T, Moheimani NR, Laird DW, Bahri PA (2019). Stepwise culture approach optimizes the biomass productivity of microalgae cultivated using an incremental salinity increase strategy. Biomass Bioenerg.

[CR24] Kim G, Bae J, Lee K (2016). Nitrate repletion strategy for enhancing lipid production from marine microalga *Tetraselmis* sp. Biores Technol.

[CR25] Kuczynska P, Jemiola-Rzeminska M, Strzalka K (2015). Photosynthetic pigments in diatoms. Mar Drugs.

[CR26] Lin Y-H, Chang F-L, Tsao C-Y, Leu J-Y (2007). Influence of growth phase and nutrient source on fatty acid composition of *Isochrysis galbana* CCMP 1324 in a batch photoreactor. Biochem Eng J.

[CR27] Maglie M, Baldisserotto C, Guerrini A, Sabia A, Ferroni L, Pancaldi S (2021). A co-cultivation process of *Nannochloropsis oculata* and *Tisochrysis lutea* induces morpho-physiological and biochemical variations potentially useful for biotechnological purposes. J Appl Phycol.

[CR28] Metting FB (1996). Biodiversity and application of microalgae. J Ind Microbiol.

[CR29] Molina Grima E, Sánchez Pérez JA, García Camacho F, Fernández Sevilla JM, Acién Fernández FG (1994). Effect of growth rate on the eicosapentaenoic acid and docosahexaenoic acid content of *Isochrysis galbana* in chemostat culture. Appl Microbiol Biotechnol.

[CR30] Napier JA, Usher S, Haslam RP, Ruiz-Lopez N, Sayanova O (2015). Transgenic plants as a sustainable, terrestrial source of fish oils. Eur J Lipid Sci Technol.

[CR31] Nielsen GL, Faarvang KL, Thomsen BS, Teglbjærg KL, Jensen LT, Hansen TM, Ernst E (1992). The effects of dietary supplementation with *n* − 3 polyunsaturated fatty acids in patients with rheumatoid arthritis: a randomized, double blind trial. Eur J Clin Invest.

[CR32] Pfaffinger CE, Schöne D, Trunz S, Löwe H, Weuster-Botz D (2016). Model-based optimization of microalgae areal productivity in flat-plate gas-lift photobioreactors. Algal Res.

[CR33] Pfaffinger CE, Severin TS, Apel AC, Göbel J, Sauter J, Weuster-Botz D (2019). Light-dependent growth kinetics enable scale-up of well-mixed phototrophic bioprocesses in different types of photobioreactors. J Biotechnol.

[CR34] Polishchuk A, Valev D, Tarvainen M, Mishra S, Kinnunen V, Antal T, Tyystjärvi E (2015). Cultivation of Nannochloropsis for eicosapentaenoic acid production in wastewaters of pulp and paper industry. Biores Technol.

[CR35] Poudyal H, Panchal SK, Diwan V, Brown L (2011). Omega-3 fatty acids and metabolic syndrome: effects and emerging mechanisms of action. Prog Lipid Res.

[CR36] Rasdi NW, Qin JG (2015). Effect of N:P ratio on growth and chemical composition of *Nannochloropsis oculata* and *Tisochrysis lutea*. J Appl Phycol.

[CR37] Rashid N, Ryu AJ, Jeong KJ, Lee B, Chang Y-K (2019). Co-cultivation of two freshwater microalgae species to improve biomass productivity and biodiesel production. Energy Convers Manag.

[CR38] Remize M, Brunel Y, Silva JL, Berthon JY, Filaire E (2021). Microalgae *n* − 3 PUFAs production and use in food and feed industries. Mar Drugs.

[CR39] Rennie KL, Hughes J, Lang R, Jebb SA (2003). Nutritional management of rheumatoid arthritis: a review of the evidence. J Hum Nutr Diet.

[CR40] Riemann B (1978). Carotenoid interference in the spectrophotometry determination of chlorophyll degradation products from natural populations of phytoplankton1. Limnol Oceanogr.

[CR41] Rodolfi L, Chini Zittelli G, Bassi N, Padovani G, Biondi N, Bonini G, Tredici MR (2009). Microalgae for oil: strain selection, induction of lipid synthesis and outdoor mass cultivation in a low-cost photobioreactor. Biotechnol Bioeng.

[CR42] Ryckebosch E, Muylaert K, Foubert I (2012). Optimization of an analytical procedure for extraction of lipids from microalgae. J Am Oil Chem Soc.

[CR43] Ryckebosch E, Bruneel C, Termote-Verhalle R, Goiris K, Muylaert K, Foubert I (2014). Nutritional evaluation of microalgae oils rich in omega-3 long chain polyunsaturated fatty acids as an alternative for fish oil. Food Chem.

[CR44] San Pedro A, González-López CV, Acién FG, Molina-Grima E (2014). Outdoor pilot-scale production of *Nannochloropsis gaditana*: Influence of culture parameters and lipid production rates in tubular photobioreactors. Biores Technol.

[CR45] Schädler T, Caballero Cerbon D, de Oliveira L, Garbe D, Brück T, Weuster-Botz D (2019). Production of lipids with *Microchloropsis salina* in open thin-layer cascade photobioreactors. Bioresour Technol.

[CR46] Schenk PM, Thomas-Hall SR, Stephens E, Marx UC, Mussgnug JH, Posten C, Hankamer B (2008). Second generation biofuels: high-efficiency microalgae for biodiesel production. Bioenergy Res.

[CR47] Sijtsma L, de Swaaf ME (2004). Biotechnological production and applications of the ω-3 polyunsaturated fatty acid docosahexaenoic acid. Appl Microbiol Biotechnol.

[CR48] Slocombe SP, Zhang Q, Ross M, Anderson A, Thomas NJ, Lapresa Á, Day JG (2015). Unlocking nature’s treasure-chest: screening for oleaginous algae. Sci Rep.

[CR49] Sun Z, Wang X, Liu J (2019). Screening of Isochrysis strains for simultaneous production of docosahexaenoic acid and fucoxanthin. Algal Res.

[CR50] Tejido-Nuñez Y, Aymerich E, Sancho L, Refardt D (2020). Co-cultivation of microalgae in aquaculture water: interactions, growth and nutrient removal efficiency at laboratory- and pilot-scale. Algal Res.

[CR51] Tocher DR (2015). Omega-3 long-chain polyunsaturated fatty acids and aquaculture in perspective. Aquaculture.

[CR52] Tocher DR, Betancor MB, Sprague M, Olsen RE, Napier JA (2019). Omega-3 long-chain polyunsaturated fatty acids, EPA and DHA: bridging the gap between supply and demand. Nutrients.

[CR53] Volker DH, Fitzgerald P, Major GA, Garg M (2000). Efficacy of fish oil concentrate in the treatment of rheumatoid arthritis. J Rheumatol.

[CR54] Wang B, Li Y, Wu N, Lan CQ (2008). CO_2_ bio-mitigation using microalgae. Appl Microbiol Biotechnol.

[CR55] Winwood R (2013). Recent developments in the commercial production of DHA and EPA rich oils from micro-algae. OCL.

[CR56] Wolf L, Cummings T, Müller K, Reppke M, Volkmar M, Weuster-Botz D (2021). Production of β-carotene with *Dunaliella salina* CCAP19/18 at physically simulated outdoor conditions. Eng Life Sci.

[CR57] Zhao P, Yu X, Li J, Tang X, Huang Z (2014). Enhancing lipid productivity by co-cultivation of Chlorella sp. U4341 and Monoraphidium sp. FXY-10. J Biosci Bioeng.

