# Supplementary material for: Simultaneous photoautotrophic production of DHA and EPA by Tisochrysis lutea and Microchloropsis salina in co-culture
Source: Bioresour Bioprocess. 2022 Dec 19;9(1):130. doi: 10.1186/s40643-022-00612-5 (PMC10991112; doi:10.1186/s40643-022-00612-5)
Supplement: Supplementary file 1 — Additional file 1: Fig. S1. Phototrophic batch process with T. lutea (●) and M. salina (●) in co-culture with an inoculation ratio of 1:3 in flat-plate gas-lift photobioreactors under simulated climate conditions. (A) cell dry weight concentration of T. lutea, (B) cell dry weight concentration of M. salina, (C) total cell dry weight concentration, (D) nitrate concentration, (E) DHA concentration, (F) EPA concentration, (G) DHA content of cell dry weight, (H) EPA content of cell dry weight. The batch process was operated at a working volume of 1.8 L, pH 8.0 and an initial nitrate supply of 3.6 g L-1. [file 40643_2022_612_MOESM1_ESM.docx]

Supplementary file

Figure 1: Phototrophic batch process with T. lutea (●) and M. salina (●) in co-culture with an inoculation ratio of 1:3 in flat-plate gas-lift photobioreactors under simulated climate conditions. (A) cell dry weight concentration of T. lutea, (B) cell dry weight concentration of M. salina, (C) total cell dry weight concentration, (D) nitrate concentration, (E) DHA concentration, (F) EPA concentration, (G) DHA content of cell dry weight, (H) EPA content of cell dry weight. The batch process was operated at a working volume of 1.8 L, pH 8.0 and an initial nitrate supply of 3.6 g L^-1^.
